# From cold chain to ambient: Benefits, risks, and evidence across cell therapy logistics

**DOI:** 10.1016/j.omtm.2025.101613

**Published:** 2025-10-11

**Authors:** John Gostage, Daniel A. Domingo-Lopez, Ruth Tarpey, Garry P. Duffy, Ruth E. Levey

**Affiliations:** 1 Anatomy and Regenerative Medicine Institute (REMEDI), School of Medicine, University of Galway, H91 W2TY Galway, Ireland; 2 Department of Anatomy, Physiology and Human Biology, School of Human Sciences, The University of Western Australia, Perth, WA 6009, Australia; 3 Instituto de Ciencia y Tecnología de Polímeros (ICTP) CSIC, 28006 Madrid, Spain; 4 Centro de Investigación Biomédica en Red de Bioingeniería, Biomateriales y Nanomedicina (CIBER-BBN), 28029 Madrid, Spain; 5 SFI Centre for Advanced Materials and BioEngineering Research Centre (AMBER), Trinity College Dublin, D02 W9K7 Dublin, Ireland; 6 CÚRAM, SFI Research Centre for Medical Devices, University of Galway, H91 W2TY Galway, Ireland

**Keywords:** cryopreservation, ambient temperature, hydrogels, medical devices, oxygen support, nutrient support, cell shipping, CAR T, cell therapy

## Abstract

With the exponential growth in the number of cell-based therapies, there is a need to look more closely at a critical factor that unites all these therapies, i.e., cryopreservation, which is currently deemed the gold standard for the storage and shipment of cells. Although cryopreservation is an integral tool in research and clinical practice, it can cause logistical issues (associated with cold chain transport), financial strain, cell dysfunction, and reduced cell viability. Many cell types that are currently being used in cell therapy, or have high clinical potential, have shown to be affected by cryopreservation. Research groups regularly highlight the need for optimized cryopreservation techniques as well as novel alternatives to avoid cryoprotectants. Here, we highlight the potential of ambient cell transport and how, by optimizing three key elements, nutrient, oxygen, and structural support, it may be possible to avoid cryopreservation during cell transport. Ambient cell transport has various benefits including circumventing ultra-low temperatures during shipment, avoidance of cryopreservation-induced cell damage, and offering a more cost-effective and accessible cell transport option. As the cell therapy field evolves, we must actively evolve with it and explore new ways to ensure effective transport of potent, viable, and efficacious cell-based treatments.

## Graphical abstract

**Figure:**
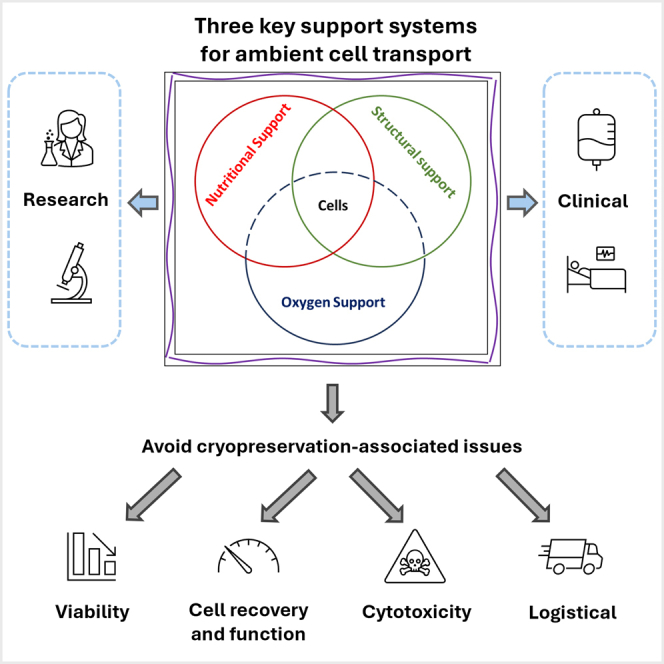

## Teaser

Levey and colleagues review the effects of cryopreservation (cellular and logistical) on cell therapies and propose ambient transport as a potential viable alternative to transport cells for clinical use and/or research. Avoiding cryopreservation in cell therapies could potentially minimize logistical drawbacks and costs while avoiding cryogenic-induced cell dysfunction.

## Introduction

Storage and shipment of cells for scientific research and clinical application are integral and indispensable. The ability to ship cells from one facility to another allows for off-site cell manufacture (for cell therapy), cell research, clinical analysis, and scientific networking. Most published research papers that used cells (*in vitro* and/or *in vivo*) for research used cells that were once shipped or stored using cryopreservation techniques. Cryopreservation is currently the gold standard for storing and shipping cells for research and clinical purposes. However, the cryogenic process can negatively affect some cell types, including cells used in clinical applications. This itself is an issue, as cell therapies need to exhibit high viability, efficacy, and potency to elicit patient benefit.^1^ According to the last gene, cell, and RNA therapy landscape report from the American Society of Gene & Cell therapies (Q1, 2025), there are 139 gene, RNA, and cell therapies (71 nongenetically modified cell therapies, 35 RNA therapies, and 33 gene therapies) approved for clinical use, and more than 4,000 therapies are in the pipeline, ranging from preclinical through pre-registration.^2^ With growing demand and approval of cell-based therapies, more research is required to fully determine the acute, long-term, and in-body effects of cryopreservation on these cells post-thaw.

Cell therapies are becoming a multibillion sector, with hundreds of deals and acquisitions signed by advanced molecular companies monthly. For instance, in 2023, the worldwide market for cell therapy reached a valuation of USD $14.52 billion. Forecasts indicate that it is set to reach USD $97 billion by 2033, with an anticipated compound annual growth rate (CAGR) of 20.9% between 2024 and 2033.^3^ With the expanding cell therapy market and its increased financial prosperity, it is not surprising that momentum is growing to streamline and optimize cell therapy practices. For example, exploring novel ways to store and ship cells: avoiding cryopreservation during the period between cell manufacture and cell therapy transfusion may yield more auspicious results in a clinical setting while simultaneously alleviating logistical pressures (cold chain transport supply) and cryo-associated cell damage and dysfunction. Here, we will highlight how cryopreservation affects some cell therapies and clinically relevant cells, summarize some of the logistical pitfalls associated with cold chain transport, and provide insights into alternative methods of cell shipment, with an emphasis on ambient shipping (in parallel with hydrogel encapsulation methods).

## The cryopreservation process and cryoprotectants

**Figure 1:**
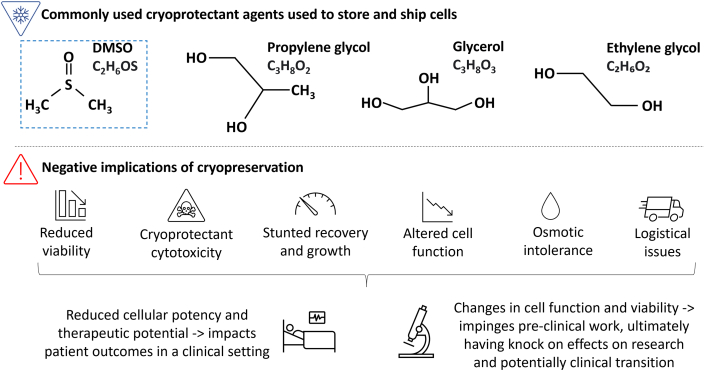
Commonly used (permeant) CPAs used in biological and clinical research DMSO (blue box) is routinely used in cryopreservation techniques for storage and shipment. Included are some examples of the negative implications of cryopreservation.

Cryopreservation is a preservative process whereby the functionality and viability of cells, organoids, and other biospecimens are safeguarded for long-term storage and/or transport. Once cells are exposed to sub-zero (below 0°C) temperatures, they are immediately at risk of freezing injury. Water is the most abundant molecule in cells, constituting 70% or more of the total cell mass. It is this attribute that poses the greatest risk during the freezing process. Freezing can lead to the formation of ice crystals intra- and extracellularly, subsequently leading to osmotic stress and mechanical damage to cell membranes and organelles, ultimately rendering the cell biophysically and biochemically impaired.^4^ To mitigate against freezing injury, an appropriate cryoprotectant (CPA) in conjunction with effective cooling and thawing rates must be implemented. CPAs can be classified as permeant and non-permeant, based on their ability to permeate the cellular membrane.^5^^,^^6^ Common permeant CPAs (Figure 1) include dimethyl sulfoxide (DMSO), glycerol, ethylene glycol (ethane-1,2-diol), and propylene glycol (propane-1,2-diol). Due to their low molecular weight and size, these permeable CPAs can penetrate cell membranes easily. These CPAs have the capacity to interact with water *via* hydrogen bonding; it is this interaction that depresses the freezing point of water and reduces the ability of water molecules to interact with themselves, thus reducing crystal formation as fewer critical nucleation sites exist. However, CPAs pose a threat to cells. For example, DMSO, the most commonly used CPA, is known to be cytotoxic to cells and, if not removed and washed away properly following cryopreservation, DMSO can cause numerous cell-specific problems, including reduced cell viability, stunted proliferation, decreased adhesion, changes in cell morphology, reactive oxygen species (ROS) production, increased apoptotic events, and cell death (Figure 1).^7^^,^^8^ Additionally, in a clinical setting, cryopreservation of cells with DMSO has caused post-transplantation complications (e.g., neurological, gastrointestinal, cardiovascular, and hepatic) due to its toxicity,^9^^,^^10^ and DMSO/CPA washing steps also add an additional burden to healthcare workers prior to infusion.

Alternative natural non-permeating CPAs (which accelerate the cell dehydration process), such as sucrose, trehalose,^11^ or low-molecular-weight hyaluronic acid (HA),^12^ have been recently investigated to reduce the need for DMSO in the cryopreservation of suspension or microencapsulated cells.^6^ These compounds have been added to many protocols, obtaining better results than with DMSO alone and allowing a decrease in the DMSO percentage in the CPA solution, reducing compound-associated toxicity. Despite providing some benefits in the extracellular environment, non-permeating CPAs do not offer primary ice protection inside the cells or overcome the risk of cell death due to intracellular ice formation.^6^

In addition to CPAs, different cooling methods can be used to reduce cell damage during cryopreservation. The most used methods are vitrification and slow freezing. Vitrification consists of the rapid freezing of the cell product (in less than 10 min) using high amounts of CPAs (40% w/v or more). In this way, the cell solution is quickly cooled below its glass transition temperature (below −130°C) without ice crystal formation, resulting in an amorphous cold viscous fluid.^6^ Despite intracellular ice crystal formation being reduced in this method, the high quantities of CPAs required are normally toxic to mammalian cells, which limits their use (Figure 1). Alternatively, in slow-freezing methods, cell samples are cooled in a controlled manner (usually −1°C/min) using lower concentration of CPAs. During this process, the formation of ice crystals in the whole sample cannot be avoided, requiring dehydration by CPAs and optimal cooling rates to prevent intracellular ice crystal formation injury.^6^

Cell therapies, including the widely acclaimed chimeric antigen receptor (CAR) T cell therapies, utilize DMSO in their manufacturing process, where cryopreservation is a crucial step in cell therapies as it extends the shelf-life and allows for long-distance shipping of the cell-based treatment. CAR T therapies frequently use DMSO at a concentration of 10% (v/v); however, some use lower levels, e.g., tisagenlecleucel and axicabtagene ciloleucel contain 7.5% and 5% DMSO, respectively.^13^ Moreover, most CAR T therapies do not remove DMSO before infusion. The Committee for Advanced Therapies (CAT) states that for clinical DMSO infusion, no more than 1% of the total plasma volume of the patient should be infused.^14^ With the risks of cryopreservation and the cytotoxic effects of CPAs, it is important to consider their ramifications in the context of cell therapies (see later subsection: the effect of cryopreservation on clinically relevant cells).

## Logistical implications of cryopreservation—cold chain transport

Cryopreservation, CPAs, and subzero temperatures are key in current cell transport methods. However, shipping cells either with liquid nitrogen (−196°C) or with dry ice (−78.5°C) pose logistical obstacles and in some cases risks (Table 1). Liquid nitrogen shipment, especially *via* air, is subject to international dangerous goods regulations (UN-No. 1977: nitrogen, refrigerated liquid [cryogenic liquid]). It is known to be hazardous, prohibitively expensive, and associated with extensive documentation and country- and courier-specific approval. Dry ice is more economical and imposes less hazards compared to liquid nitrogen shipment—if used appropriately, dry ice can maintain its temperature for 24–72 h.^27^^,^^28^ However, a pitfall with dry ice is that it sublimates rapidly if the wrong quantity or packaging is used. As a result, dry ice is classed as a hazardous material because of its potential to cause an explosion and suffocation, hence, dry ice shipments must display a hazard class 9 label (UN No. 1845) on the packaging.^27^ Based on recent Federal Express shipping information, more than 50% of countries prohibit delivery with dry ice. A list of countries affected by restricted dry-ice import can be found at https://www.fedex.com/en-us/service-guide/dangerous-goods/international-locations.html.^29^ However, many of these countries have limited research infrastructure and typically spend less than 1% of their GDP on research and development.^30^

**Table 1:** Comparison of logistical issues associated with cryopreserved and ambient cell shipment

	Cryopreserved	Ambient transport	References
Product preparation (freezing protocol and formulation)	• CPA exposure • optimization of freezing process • can take numerous days for cells to safely reach desired super-low temperatures • appropriate packaging and means to maintain super-low temperatures (e.g., dry ice) required	• NO CPA exposure • NO pre-shipment freezing process required • cell can be prepared easily and relatively quickly for shipment, e.g., cells removed from incubator, counted, resuspended in transporting solution (and/or gel), and transferred to specialized shipment device/vessel	Fuller et al.^15^; Meacle et al.^16^
Maintenance of correct shipment temperature and conditions	• ultra-low temperature needs to be maintained—temperature sensors required and replenishment of cold source may be required	• for general ambient conditions no temperature control required • for fixed ambient temperature, specialized packaging (e.g., Crēdo Cube [PELICAN BioThermal, Plymouth, USA]) and temperature sensors may be required	Fuller et al.^15^; Pogozhykh et al.^17^; Stacey et al.^18^; Meneghel et al.^19^; Meacle et al.^16^
Integrity of vialed products	• vial integrity at risk of being compromised due to super-low temperatures	• ambient temperatures do not pose a risk to vial/vessel integrity	Zuleger et al.^20^; Meacle et al.^16^
Radiation risk	• risk of radiation exposure	• risk of radiation exposure	Meacle et al.^16^; Petzer et al.^21^
Supply chain disruptions	• risk of being affected by supply chain disruptions—extended delays can lead to a risk of super cold temperatures not being maintained, subsequently leading to cell thawing and CPA exposure	• risk of being affected by supply chain disruptions—hold-ups can influence exposure to different ambient conditions. Extended delays could lead to undesirable cell expansion and/or the depletion of nutrient and oxygen support	Meacle et al.^16^; Brenner^22^; PELI Biothermal^23^
Delivery, thawing and subsequent use	• package needs to be processed quickly upon delivery to minimize risk of cell thawing • some countries may not accept delivery of dry-ice or liquid nitrogen cell shipments • CPA washing may be required • ultra-low freezer may be required to continue storage before use • cell expansion may be required to help with acclimatization post-thaw and/or increase viable cell count	• NO CPA washing required • may require removal of structural support (e.g., hydrogel) • testing cell viability and/or functionality may be required • minimal manipulations at delivery point—ambient transport has the potential for cells to be quickly moved to culture in recipient lab or used immediately in an experimental or clinical setting	Meacle et al.^16^; Baboo et al.^24^
Environmental impact	• maintenance of super-low temperatures *via* use of liquid nitrogen or dry ice will have an environmental impact; for example, 1 kg dry ice is computed to produce approximately 0.5466 m^3^ of CO_2_ gas at 21.1°C. Mitigatory action may be required	• maintenance of super low temperatures is not required during ambient shipment, subsequently reducing the environmental impact	Belkhir and Elmeligi^25^; Ajala et al.^26^

Effective logistical planning is key to delivering superior quality cryopreserved products in scientific and clinical settings. However, it is widely acknowledged that limitations still exist within cold chain transport.^15^ See Table 1 for a summary of logistical issues associated with cryopreserved cell shipment and how these are compared to non-cryopreserved, i.e., ambient cell transport. Listed below are logistical considerations regarding cold chain transport and some associated risks:

(1) Product preparation (freezing protocol and formulation): before shipment even initiates, cells must be correctly prepared, frozen under optimized conditions (i.e., freezing rate and freezing solution), appropriately stored in the correct vial, and transferred to a suitable shipment container. If these parameters are not carried out effectively, then the biological products will be already entering cold chain in a sub-optimal capacity.^15^
(2) Maintenance of correct shipment temperature and conditions: it is widely recognized that correct cryogenic temperatures must be maintained to maximize viability and functionality of cells.^17^^,^^18^^,^^19^ However, with ultra-low cryogenic temperatures being most desirable, they also come with drawbacks including energy consumption to maintain low temperatures, coolant quantity and phase, effective temperature monitoring, external temperature influences, and most notably cost.^15^
(3) Integrity of vialed products: cryogenic temperatures can potentially compromise the container closure integrity of some vials, subsequently potentially exposing the contents to contamination and/or a build-up of CO_2_ or N_2_ (which can also influence the pH of the sample) in the vial.^16^^,^^20^
(4) Radiation risk: although transport-associated radiation exposure is presumed to have a negligible effect on cell products, it still must be highlighted, as there is a lack of rigorous exploration of this area.^16^ Radiation could potentially affect cells and/or larger multicellular units (e.g., organoids or organs) at two points during transit, i.e., cosmic radiation during flight and X-ray exposure during security screening. One study showed that repeated X-ray exposure from airport hand-luggage control systems did not harm hematopoietic stem cells.^21^ However, with the growing use of flight-based transport for global cell therapies, there is a lack of thorough analysis of the effects of cosmic radiation on cell integrity and function.
(5) Supply chain disruptions: due to the global complexity of cold chain transport, numerous extraneous factors can influence supply chain, from staffing issues to custom hold-ups.^22^ It is estimated that the pharmaceutical industry loses approximately USD $35 billion a year due to failures in temperature-controlled logistics.^23^ These issues must be mitigated by ensuring cryogenic conditions are maintained in the result of a logistical hold-up.
(6) Delivery, thawing, and subsequent use: upon delivery, cells and/or biological products must be processed quickly. If products are left unattended and in the wrong storage conditions, it could impinge on their viability and function post-thaw. Furthermore, the correct thawing rate must be implemented and any additional post-thaw steps (e.g., CPA removal) must be executed,^24^ adding additional manipulation steps and burdening clinical or research staff. If any of these final steps are not properly implemented, it could render all previous stages nullified.
(7) Environmental impact: the pharmaceutical sector is far from being green, producing 55% more CO_2_ emissions compared to the automotive sector.^25^ Transporting cells and other biological products contributes to high carbon emissions. With growing concerns, some institutes require “carbon offset solutions,” which provoke additional financial investment and planning.

## The effect of cryopreservation on clinically relevant cells

**Figure 2:**
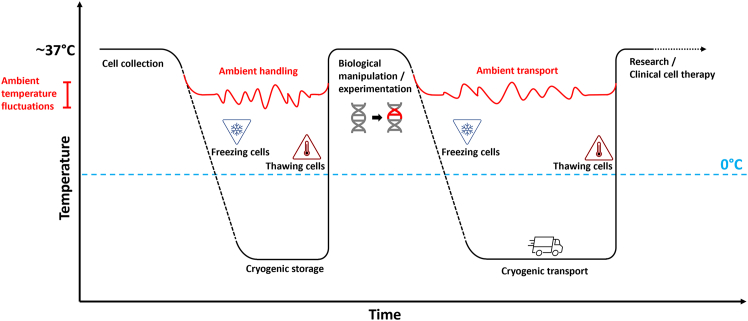
Temperature changes over time during routine cell harvest, processing, and transport for clinical cell therapy or research Up to two freeze-thaw cycles occur during the cell therapy manufacture and delivery process, ultimately allowing for extended “shelf-life” and greater flexibility and feasibility during the cell therapy process. Cryopreservation is primarily used during cell transport from the manufacturing facility to the clinical setting for patient infusion. Included in the schematic are points in which ambient transportation methods can be potentially integrated into the cell therapy pipeline. Ambient transport solutions avoid cryopreservation and reduce the number of manipulations, subsequently reducing the risks of CPA exposure, improving logistical planning, and minimizing costs. Novel ambient transportation methods could be key in the immediate handling and/or transport of freshly harvested samples, e.g., post-apheresis or post-umbilical cord sample collection.

Cell therapies are being continuously developed and refined—since records began in 1986, over 5639 interventional cancer cell therapy clinical trials have been registered in mid-2024.^31^ In the United States alone, it is predicted that 40–50 new cell and gene therapies will be launched by 2030, half of which expected to target B cell (CD-19) lymphomas and leukemias.^32^ Projections suggest approximately 93,000 American patients will be treated in 2030 with cell and gene therapies, generating roughly USD $24.4 billion.^33^ The cell and gene therapy market is growing at an exponential rate; however, virtually all cell therapies utilize the cryopreservation process at different stages of their application (Figure 2). Here, we discuss the potential negative implications of cryopreservation on key cell therapies and a few examples of clinically relevant cell types. Where possible, we will show comparisons between cryopreserved vs. “fresh” (no cryo-intervention) regimes. Adding to this, Table 2 summarizes the potential negative effects of cryopreservation on these cell types and other clinically relevant cell types.

**Table 2:** Cell types that have clinical application or high clinical translational potential within cell therapy fields and the main effects of cryopreservation on these cell types

Cell line	Medical relevance	Effect following cryopreservation	References
Pancreatic islet cells	islet cell transplantation for treating diabetes and hyperglycemia and providing insulin independence endocrinology and diabetology research	• native islets are multi-cellular structures; due to this, they exhibit non-uniform water and CPA content at different levels of their structure (core vs. outer later) during cryopreservation. • after exposure to the freezing and thawing, pancreatic islets are at a higher risk of being either non-functional, apoptotic, or non-viable • different CPAs and cryopreservation techniques induce varying (low-to-moderate) viability levels post-thaw • reduced functionality post-thaw (e.g., mitochondrial membrane potential and ATP production) • conventionally cryopreserved islets failed to normalize blood glucose levels in streptozotocin-induced diabetic mice	Marquez-Curtis et al.^34^ Kojayan et al.^35^ von Mach et al.^36^ Zhan et al.^37^
Hepatocytes	liver-based metabolic diseases and liver failure	• reduction in viable cells • reduction in ATP levels • reduced mitochondrial membrane potential • human and mouse hepatocytes exhibit reduced oxygen consumption rate post-thaw (compared to fresh) • activation of apoptotic pathways	Stéphenne et al.^38^ Iansante et al.^39^ Chow-Shi-Yée et al.^40^
Sperm	*in vitro* fertilization and andrology research	• increased ROS production • membrane impairment • DNA fragmentation • reduced viability • apoptotic signaling • reduced or stunted motility • cytoskeletal impairment • reduced fertilizing potential • mitochondrial impairment • epigenetic changes	Estudillo et al.^41^
Oocyte	*in vitro* fertilization and gynecology research	• increased ROS production • membrane impairment • DNA fragmentation • reduced viability • apoptotic signaling • epigenetic changes • impaired Ca^2+^ signaling • mitochondrial impairment • altered lipidic profile	
MSCs	frequently used in regenerative medicine, used to treat various pathologies, including bone and cartilage diseases, neurological disorder, diabetes, and cardiac ischemia	• reduced viability (highly variable; many factors can influence viability, including CPA used, freezing protocol, donor profile, and source) • altered immunomodulatory potential • altered migration/homing potential • DMSO CPA may influence cell-cycle and chromosomal stability (altered karyotype increased cancer risk) • DMSO CPA affects DNA integrity, apoptosis, cell cycle, and function of human BM-MSCs	Wang and Li^42^ Ding et al.^43^
Hematopoietic stem cells	a wide range of applications, including a variety of malignant and non-malignant diseases, some cancers, blood disorders, and immune deficiency disorders	• reduced viability (highly variable; many factors can influence viability, including CPA used, freezing protocol, donor profile, and source) • cellular apoptosis and delayed cell engraftment (if exposed to DMSO CPA for too long) • altered functionality	Cottle et al.^1^; Hornberger et al.^44^
T cells	CAR T cell therapies have been used to treat hematological malignancies, e.g., lymphoma, leukemia, and multiple myeloma	• varied recovery rates post-thaw • reduced viability • potential (CI)DOCD effects • altered phenotype analysis and anti-tumor reactivity	Brezinger-Dayan et al.^45^
Natural killer (NK) cells	NK cells initiate innate immune responses toward tumor and virus-infected cells; hence, they are becoming an attractive candidate for the treatment of cancers, viral infection, and some autoimmune conditions	• reduced viability • poor expansion post-thaw—reduced viability over time (unable to salvage with interleukin-2 [IL-2] supplementation) • immunophenotype changes, e.g., activating receptor NKG2D was decreased post-thaw • some short-term effects on cytokine production (e.g., tumor necrosis factor α [TNF-α]) • reduced cytolytic activity • impaired motility	Saultz and Otegbeye^46^

### Chimeric antigen receptor T cells

CAR T cell therapy is a form of immunotherapy in which patients’ T cells are genetically modified to target specific antigens (e.g., CD19) on the surface of cancerous cells. CAR T cell therapy has proven to be an effective treatment for blood cancers, including lymphomas, some forms of leukemia, and, more recently, multiple myeloma. The following Food and Drug Administration (FDA)-approved CAR T cell therapies are currently available: Kymriah (tisagenlecleucel), Yescarta (axicabtagene ciloleucel), Tecartus (brexucabtagene autoleucal), Breyanzi (lisocabtagene maraleucel), Abecma (idecabtagene vicleucel), Carvykti (ciltacabtagene autoleucel), and most recently (November 2024) Aucatzyl (obecabtagene autoleucel).^47^^,^^48^ Most CAR T cell therapies require cryopreservation for cell storage and shipment (Figure 2), as cell manufacturing sites are not commonly on-site.^49^ Consequently, a question that arises among clinicians and scientists is: “does cryopreservation dampen the potency, viability, and/or functionality of CAR T cell therapies?” A key CAR T cell therapy quality and regulatory parameter is the percentage of viable cells (expressing the CAR) infused into a patient. A required infusion level of ≥80% viable CAR T cells has been set by the FDA. However, in clinical trials (e.g., tisagenlecleucel) and in some commercial settings outside of the United States, the viability threshold is lower at ≥ 70%. The FDA has indicated that non-viable CAR T cells pose a potential clinical safety risk.^50^ Moreover, due to the cytotoxic effects of CPAs, CAR T cell therapies must be administered to a patient rapidly, normally within 30–90 min post-thaw. Prolonged exposure to CPAs could negatively influence the biophysical and biochemical properties of the manufactured cell therapy.^51^^,^^52^

In some CAR T cell manufacturing facilities, particularly those utilizing the Miltenyi Prodigy device, it has been reported that CAR T cells exhibit viability as low as 47.2% post-thaw (range: 47.2%–68.9%), which is considerably lower than the FDA-required level.^53^ Brezinger-Dayan et al. showed CAR T cell recovery post-thaw was 67% (range: 56%–91%). When compared to fresh, cryopreservation did not affect the frequency of CAR+ T cells, CD3 T cells, CD4/CD8 subpopulation, or their capacity to expand; however, there was a reduction in the recovery rate of cells following cryopreservation. But ultimately, cryopreservation did not affect the clinical outcome.^45^ Reduced post-thaw CAR T cell recovery, as low as ∼50% (mean: 76.7%), was observed in another study—the authors recommended cryopreservation post-manufacture may be preferable to cryopreserving the starting fraction.^54^ Shah et al. showed a marked difference in viability of fresh vs. cryopreserved anti-CD19/CD20 CAR T cells. Mean cell viability at infusion was 93% for fresh (*n* = 14), compared to 63% for the cryopreserved group (*n* = 5). Complete response rate was 79% vs. 40%, respectively (not statistically significant). Low sample sizes must be acknowledged.^55^ Dreyzin et al. showed that when compared to pre-cryopreservation and fresh cell samples, CD22 and CD19/22 CAR T cell viability was significantly reduced post-cryopreservation (CPA used: 5%–10% DMSO); however, the median CAR T viability at infusion remained higher than 80%. Cryopreserved anti-CD22 CAR T cells exhibited no differences in disease response, incidence of toxicities, *in vivo* expansion, or persistence when compared to patients receiving fresh anti-CD22 CAR T cells. However, compared to fresh, cryopreserved anti-CD19/22 CAR T cells exhibited a reduction in persistence in patients bone marrow (BM) at day 28. Furthermore, lower viability correlated with lower day 28 persistence; these observations highlight the potential of delayed or long-term effects of “viable” cells post-thaw.^49^ Ultimately, this study highlighted the equal patient benefit of both fresh and cryopreserved anti-CD22 and bispecific anti-CD19/22 CAR T cells for B cell leukemia treatment.

Panch et al. explored the effect of cryopreservation on peripheral blood mononuclear cells (PBMNCs) and CAR T cell characterizations. Cryo-thawed PBMNCs (*n* = 29) used to generate (CD19) CAR T cells saw a significant (*p* = 0.003) reduction in viability compared to fresh (*n* = 27). Maximum levels of peripheral blood T cells expressing CD22 CAR were significantly reduced in cryopreserved products compared to fresh, indicating reduced *in vivo* persistence. Moreover, cryopreservation of PBMNCs and/or of the final manufactured CAR T cells did not impact transduction efficiency, fold expansion, the proportion of T cell subsets (including naive, effector, and central memory T cells), CD3%, or CD4:CD8 ratios at harvest or pre-infusion, respectively. Hence, this investigation supports cryopreservation as a viable strategy in CAR T cell manufacture.^56^ However, the authors highlighted delayed onset cell death (DOCD) and the implications and importance of investigating this phenomenon further in CAR T cell therapy. DOCD and more specifically cryopreservation-induced (CI)DOCD remains a significant drawback among cryobiologists, as these concerns extend to all cell therapies.^57^ Due to the immediate infusion of cells post-thaw, it is very difficult to determine DOCD in an *in vivo* setting and hence determine the “true” cell survival number. DOCD usually occurs 6–48 h post-thaw, driven by activation of apoptotic signaling (notably proteolytic activation of caspase-3), but for CAR T therapies, it may arise within a couple of hours to days.^56^^,^^58^ CIDOCD in CAR T (and other) cell therapies warrants rigorous exploration, as this could help understand *in vivo* cell functionality post-thaw, subsequently assisting with dose and delivery optimization.

More recently, Cadinanos-Garai et al. showed cryopreservation had minimal impact on CAR T cell phenotype and functionality. Day 10 cryopreserved products retained phenotypic profiles similar to fresh samples, showing only slight changes in subset frequencies (decreased Th1 or Tc1 and increased CD4^+^ AN cells) and modest alterations in CD69, HLA-DP, DQ, DR, TIM3, or GLUT1 expression—levels of exhaustion, senescence, apoptotic, and cytolytic markers remained overall stable. Memory phenotypes and antigen responsiveness remained largely preserved post-cryopreservation. Data from this study aligns with other clinical investigations (previously discussed:^49^^,^^56^), showing comparable efficacy between cryopreserved and fresh CAR T cell products.^59^

With some discrepancies between cell viability thresholds for cell therapy infusion, i.e., ≥70% or ≥80%, an investigation by Chong et al. set out to explore these effects of viabilities lower than 80% (but higher than 70%) vs. 80% or higher in tisagenlecleucel cell therapy products. It was found that CAR T product viability between 70% and 80% did not affect clinical outcomes (progression-free survival and overall survival) for pediatric and young adult B cell acute lymphocytic leukemia (ALL) and adult diffuse large B cell lymphoma (DLBCL). For ALL, 15/123 (12%) products had less than 80% viability (median: 77%, range: 56%–79.8%), and 4/25 (16%) products for DLBCL had less than 80% viability (median: 75.1%, range: 73.7%–79.9%). Moreover, regarding the tisagenlecleucel product, there is no evidence to support differences in CAR T cell expansion or survival *in vivo* between patients receiving CAR T cells with <80% (but higher than 70%) viability compared with those receiving CAR T cells with ≥80% viability. There was no statistically significant association between cell product viability and complete remission rate; hence, this study supports current manufacturing practices, i.e., use of cryopreservation strategies.^60^

Even though some studies observe a reduction in CAR T cell viability post-thaw, clinical outcomes and response rates overall were not significantly different when comparing cryopreserved vs. fresh. Su et al. showed that patients receiving fresh CAR T products exhibited higher incidence of acute hematological toxicity (including anemia, thrombocytopenia, and hypoleukemia), when compared to cryopreserved CAR T products.^61^ This study highlights that in some cell therapy scenarios cryopreserved cell products may be a better candidate compared to fresh cell products. Cryopreservation is standard practice in numerous CAR T therapy pipelines; it would be difficult to implement change now in something which has been optimized and investigated internationally. When weighing up the pros and cons of cryopreservation in a clinical setting, i.e., what is best for the patient, the benefits of cryopreservation for CAR T storage, transport, and pipeline efficiency currently outweigh the negative effects of the process, e.g., reduced viability upon infusion. However, down the road, the effects of CIDOCD and advancements in logistical and shipping options may encourage the use of novel cryo-free options in the CAR T pipeline. But for now, cryopreservation is key in CAR T cell therapy, and cryo-avoidance may be better suited for other established and/or novel cell therapies (Table 2).

### Pancreatic islets

Pancreatic islets are complex structures; they comprise five major endocrine cells, including α-cells (glucagon synthesis), β-cells (insulin and amylin synthesis), δ-cells (somatostatin synthesis), γ-cells (pancreatic polypeptide synthesis), and ε-cells (low in number, ghrelin synthesis), collectively working to orchestrate metabolic homeostasis.^62^ Since initial proof-of-concept studies of islet transplantation in the 1970s (laboratories of Paul Lacy and Clyde Barker), momentum has grown regarding the development of new cell therapies for insulin-dependent diabetes and associated islet dysfunction.^63^ In a historic event, FDA approved Lantidra (donislecel) in June 2023, the first allogeneic (donor) pancreatic islet cell therapy for adults with type 1 diabetes.^64^ More recently, Zimislecel (formerly VX-880), Vertex’s stem-cell-derived islet therapy with standard immunosuppression, is progressing well in clinical trials and demonstrating promising results for the treatment of type 1 diabetes.^65^

Islet transplantation offers a new option for the treatment of diabetes. However, extended islet culture has shown to negatively affect cell viability and endocrine function; hence, cryopreservation has been suggested as a potentially beneficial tool to improve feasibility.^66^^,^^67^ Unfortunately, a major pitfall that has hindered the progression of islet therapy research area is the negative effect of conventional cryopreservation practices (Table 2). Various studies over the last few decades have shown reduced islet viability post-thaw; for example, Langer et al. showed that conventional cryopreservation reaped 51.8% ± 3% viability, whereas fresh islets had 85.6% ± 1.4%.^68^ Von Mach et al. demonstrated 22.0% ± 0.9% islet viability with 5% DMSO, 49.5% ± 1.3% with 10% DMSO, 60.5% ± 1% with 14.4% DMSO, 19.6% ± 1.3% with 18.3% DMSO, and 8.6% ± 0.8% with 21.7% DMSO.^36^ Other conventional cryopreservation techniques have displayed 59.1%,^69^ 50%,^70^ and 59.1%–62.2%^37^ islet cell viability post-thaw. Post-thaw also impedes islet cell functionality; for example, conventionally cryopreserved islet cells showed a significant reduction in ATP production and mitochondrial membrane potential of porcine, mouse, and human islet cells compared to live controls. Furthermore, following transplantation of islet cells into streptozotocin-induced diabetic mice, conventionally cryopreserved islets failed to normalize blood glucose levels in all recipients (even with increased numbers of islets).^37^ These studies indicate that conventional cryopreservation techniques are potentially not a feasible option to store or transport pancreatic islet cells.

Over the last several decades, attempts have been made to optimize islet cell cryopreservation to improve their potency, efficacy, and survival rates, with the aim of translating and utilizing them in a clinical setting. Different cooling times (0.3°C–70°C/min) in conjunction with different CPA concentrations have been trialed, but with issues persisting, further optimization and novel preservation approaches have been developed.^35^ Vitrification techniques have shown promising results in islet cell preservation, although optimization of techniques and *in vivo* translation are ongoing. Sasamoto et al. compared conventional cryopreservation against vitrification methods in rat islets. Compared to fresh islets, which demonstrated 93.2% viability, conventional cryopreservation methods yielded 67.5%–75.0% viability post-thaw, whereas quick-rate vitrification of islets using EDT324 yielded 85.8% viability post-thaw, which was significantly higher than all other cryopreservation methods tested. Comparatively, other vitrification solutions, including cryoloop (small nylon loop used to vitrify), solid-surface vitrification, and ESF40, resulted in low numbers of viable islets post-thaw—63.8%, 45.0%, and 41.2%, respectively. Regarding insulin secretory activity, fresh islets exhibited a stimulation index of 7.0 ± 1.6 μg insulin/μg DNA, whereas quick-rate freezing using 10% DMSO resulted in a stimulation index of 2.9 ± 1.2 μg insulin/μg DNA post-thaw. Moreover, compared to 10% DMSO, EDT324 significantly increased insulin secretory activity (6.4 ± 0.6 μg insulin/μg DNA) post-thaw. Furthermore, streptozotocin-induced diabetic rats transplanted with islets vitrified with EDT324 had similar glycemic values at all measured times compared with recipients of non-frozen grafts (euglycemia achieved 2 days after transplantation), whereas rats receiving DMSO cryopreserved islets exhibited significantly higher blood glucose compared to the control group at earlier time points (euglycemia achieved 6 days post-transplantation).^71^

Other studies have demonstrated novel vitrification protocols for islet preservation, for example, hollow fiber vitrification,^72^ Cryotop vitrification,^70^ nylon mesh vitrification,^73^ silk fibroin sponge vitrification,^74^ copper dish cooling and convective warming, copper dish cooling and laser nanowarming, and convective cooling and warming using a Cryotop device.^37^ However, in all instances, viability and functionality were lower than fresh islets. Moreover, in most cases, protocols must be meticulously executed using specialized equipment. From extensive literature research and to the best of our knowledge, no published technique has achieved effective islet cryopreservation with high islet functionality, viability, recovery, and subsequently clinical scalability. Hence, in the hope that one day islet cell therapy is readily available, it is important to understand the hazards of cryopreservation, so alternative options can be developed to store or transport cells while eliminating cryo-associated cell damage (Table 2).

Apart from the problems related to cryopreservation, islet transplantation is highly limited by the shortage of human islets or other insulin-producing cells available for transplantation. Due to a scarcity of human donor pancreases, less than 0.5% of type 1 diabetes patients are treatable with islet transplantation, as large quantities of islets (2–3 pancreases) are required to achieve insulin independence.^75^^,^^76^ Fortunately, advances in stem cell technology and therapeutics provide alternative islet sources: Human pluripotent stem cells (hPSCs), including embryonic (ESCs) and induced pluripotent stem cells (iPSCs), can be expanded indefinitely in culture and subsequently induced to differentiate into any cell type found in the body.^77^^,^^78^ These capabilities have motivated much interest into these cells as a potential renewable source of stem-cell-derived islets (SC-islets) for diabetes cell replacement therapy. However, the use of stem-cell-derived insulin-producing cells results in poor survival and a reduction in glucose-mediated insulin secretion.^79^^,^^80^ For instance, insulin-producing cells derived from adipose-tissue-derived stem cells (ADSCs) frozen by three different methods—(1) immediately frozen at −80°C, (2) frozen at −80°C in a BICELL freezing container, and (3) vitrification in a Cryotop device—showed a dramatic decrease in their viability in all cryopreservation groups, to 13.3%, 6.3%, and 15.6%, respectively. Additionally, cryopreservation notably decreased insulin secretion for all groups. Initially, the average insulin secretion in response to glucose stimulation stood at 57.9 pmol/L. Following cryopreservation, this dropped to 44.1 pmol/L in the −80°C group, 39.8 pmol/L in the BICELL group, and 42.8 pmol/L in the Cryotop group.^80^ A recent review by Hogrebe et al. provides an insightful overview of developments in SC-islet replacement therapy for treating type 1 diabetes. The authors note that effective cryopreservation of SC-islets is key to upscaling their clinical and market potential.^81^

### Mesenchymal stem cells

Mesenchymal stem cells (MSCs) are gaining huge traction within clinical parameters, with many MSC products receiving regulatory approval in the last 15 years.^82^ Due to the multipotent nature of MSCs, they can differentiate in a multilineage fashion and hence assist in a myriad of pathological conditions, subsequently making them a very attractive cell therapy option. MSCs can be sourced from various tissues, e.g., adipose and BM. Dependent on the source, MSCs exhibit varying differential capacity; furthermore, factors like culture conditions, passage number, delivery method, donor age, and host receptivity markedly affect MSC efficacy and potency.^83^ Here, we will briefly highlight some of the effects of cryopreservation on BM-derived MSCs (Table 2), given that they are the most sourced MSC (due to their high differential capacity).

A recent investigation by Ding et al. showed that cryopreservation of human BM-MSCs using 10% DMSO resulted in a significant ∼10% reduction in viability and ∼20% reduction in recovery rate post-thaw (compared to fresh cells). Proliferation was significantly reduced 24, 48, and 72 h post-thaw. Furthermore, compared to fresh controls, DMSO cryopreservation increased BM-MSC apoptosis, DNA damage, and ROS production and reduced the migratory ability of BM-MSCs.^43^ In another study, BM-MSCs from three young human donors showed a significant reduction in surface markers CD60 and CD105, 24 h post-thaw (compared to fresh controls). However, the degree of surface marker expression was different between donors, highlighting individual changes in response to cryopreservation. The same study also reported significant reductions in viability, metabolic activity, adhesion potential, and colony-forming unit fibroblast ability of BM-MSCs up to 24 h post-thaw.^84^ A systematic review summarized some of the effects of cryopreservation on BM-MSCs across 41 published resources (26 human studies). It was noted that BM-MSC proliferation, morphology, differentiation, and immunophenotyping did not deviate from the norm following cryopreservation. However, BM-MSC viability/apoptosis, attachment, immunomodulation, and metabolism were shown to be commonly affected by cryopreservation.^7^

### Hepatocytes

Hepatocytes exhibit high regenerative capacity; hence, they are an attractive cell type for the treatment of liver diseases and to provide alternative options to orthotopic liver transplantation.^85^ Hepatocyte transplantation has been demonstrated in mouse models.^86^^,^^87^ To date, human hepatocyte transplantation has been attempted in several liver diseases, including glycogen storage disease type 1, urea cycle disorders, severe infantile oxalosis factor VII deficiency, phenylketonuria, infantile Refsum disease, and acute liver failure, all of which resulted in partial correction. The safety and short-term efficacy of clinical hepatocyte transplantation have been proven; however, some obstacles, such as long-term potency and engraftment, remain.^39^^,^^88^ Moreover, the therapeutic potential of hepatocyte transplantation is high—recently, a new allogeneic hepatocyte transplantation solution named LyGenesis (LYG-LIV0001) gained FDA clearance to begin phase 2a trial for end-stage liver disease.^89^

A major concern regarding hepatocyte cell therapy is cryopreservation. Although numerous hepatocyte cryopreservation protocols have been described,^90^^,^^91^^,^^92^^,^^93^ many published papers and research groups report persisting issues following post-thaw (Table 2). A study by Ostrowska and colleagues compared freshly isolated human hepatocytes against cryopreserved human hepatocytes. Cryopreservation significantly reduced viability, plating (attachment) efficiency, and intracellular ATP content.^94^ Reduced viability and plating efficiency of cryopreserved human hepatocytes compared to fresh hepatocytes have also been observed in other studies.^95^^,^^96^ Cryopreservation (using DMSO) of rat hepatocytes was shown to significantly promote apoptotic pathways.^40^ Refining cryopreservation and storage techniques will be critical in scaling up hepatocyte-based cell therapy. New approaches to provide an “off-the-shelf” bioartificial liver system are being continuously explored, highlighting the genuine drive and interest in this research area.^97^

## Circumventing conventional cryopreservation techniques for cell shipment

Cell transport mainly utilizes cryopreservation techniques, which pose various CPA and cryo-associated issues (Figures 1 and 2), logistical/cold chain pitfalls (Table 1), and altered cell function post-thaw (e.g., low viability, stunted growth, and altered cell functionality). Due to these issues and the evolvement of cell therapies, there is a growing interest in research attempting to trial new CPAs and/or cryopreservation techniques or move away from CPAs and cryopreservation altogether.

### Novel and non-conventional CPA and cryopreservation alternatives

Several alternatives to conventional cryopreservation have been explored, including dry preservation,^98^ hypothermic preservation,^99^ and novel hydrogel-based^100^^,^^101^ or solution-based encapsulation systems.^102^^,^^103^ Currently, solution-based and hydrogel-based systems have received the most interest. The versatility and adaptability of the materials involved in these systems could be the catalyst in progressing CPA-free storage and shipping protocols.

Solution-based systems using osmolytes such as sugars, sugar alcohols, and amino acids can help maintain the stability of biological systems subject to hard conditions, offering an alternative to cytotoxic CPAs. These solutions can be used in combination with classic CPAs or on their own. For instance, it was demonstrated that by the combination of multicomponent osmolyte solutions containing sucrose, glycerol, and/or isoleucine, it was possible to achieve the same cell recovery to that observed using 10% DMSO, for Jurkat T cells.^104^ Optimization of the concentration and content of these osmolyte solutions can induce changes in cell recovery (e.g., higher concentration of glycerol or interaction between glycerol and isoleucine with glycerol can increase cell recovery).

Trehalose, a naturally occurring glucose disaccharide with osmolyte properties, has shown promise as an alternative to conventional CPAs in cryopreservation processes.^105^ Trehalose is a non-permeant CPA and can form hydrogen bonds with biomolecules, subsequently creating a persistent glassy matrix that suspends intracellular metabolic processes during water loss. This property makes it effective in long-term cryopreservation, even in small amounts alone or combined with DMSO. The addition of trehalose to cryopreservation solutions has significantly enhanced the survival rates of various cell types, including hepatocytes, red blood cells, sperm, and oocytes, to name a few.^106^ Alternatively, trehalose alone has shown superior long-term preservation capacity for adipose tissues, compared to simple cryopreservation methods, due to its ability to maintain cell vitality and functionality.^107^ Further research is needed to optimize trehalose-based cryopreservation techniques, but its nontoxic nature and potential for single-agent use make it an attractive option in the field. On the other hand, due to their ability to protect cells from apoptosis, trehalose-containing solutions have been extensively studied as an alternative to cryopreservation, for hypothermic storage. Trehalose-containing solutions were found to be effective in preserving MSCs at 4°C, maintaining their viability above 70% for up to 3 weeks. Moreover, trehalose-preserved MSCs exhibited similar functional features (growth kinetics, expression profile of cell surface antigens, and differentiation potential) as freshly harvested cell analogues.^102^ Recently, Fuenteslópez et al. optimized trehalose delivery (*via* ultrasound in the presence of microbubbles) to MSCs. This method was able to preserve cell viability, membrane integrity, and most importantly multipotency, subsequently offering an alternative to conventional CPAs.^108^

### Avoiding CPA and cryopreservation—ambient cell shipment

**Figure 3:**
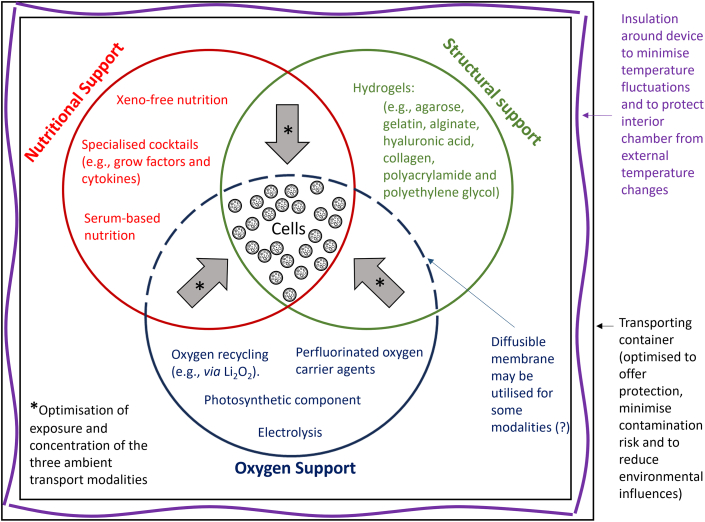
Design considerations for an ambient transport solution—three major modalities must be considered: structural, nutritional, and oxygen support Optimization stages will ultimately determine the best way in which these three modalities can interact to ensure cell health is maximized. Modality concentration, exposure time, and interaction (e.g., *via* use of a diffusible membrane) can be modified for different cell types to ensure high cell viability is maintained during ambient transport.

Due to logistical obstacles (Table 1), CPA cytotoxicity, and cell damage (Figure 1), cryopreservation may not be a prudent choice for transporting some cell types prior to clinical application. In current practice, some residual CPAs from cryo-transported cells are infused into patients receiving cell therapy. Although, a recent trend has seen a gradual reduction in CPA percentage (v/v) used in cell therapy cryo-techniques.^109^^,^^110^ Moreover, some patients receiving DMSO-cryopreserved cells experience adverse reactions or significant complications, attributable to CPA exposure.^111^ Efforts to remove/wash DMSO prior to patient infusion can lead to various issues: (1) cell loss, (2) delayed infusion time, (3) additional support staff/expertise required, and (4) risk of extended exposure of thawed cells to CPAs. Furthermore, FDA guidelines instruct minimal manipulations must be taken prior to patient infusion.^111^^,^^112^ Another important factor to also note is that DMSO has the propensity to leach and etch plastic transfusion tubing and other clinical containers, thereby heightening the risk in cGMP processes.^113^ With these factors in mind, and the exponential growth in cell therapy products, novel methodologies to avoid CPA exposure and cryopreservation must be explored: “what if at the end of the cell manufacturing process, cells could be transferred into a medical device, safely secured/encapsulated, supplied with the correct nutrients and oxygen, and shipped to the patient at ambient temperature, all without the need of cryopreservation?” This workflow suggestion circumvents cryopreservation, avoids cold chain transport, minimizes pre-infusion manipulations, and avoids CPA exposure (Figures 2 and 3). Cryopreservation is considered the gold standard method for cell transport; however, it may not be the “golden ticket” for some cell therapies.

There exists a growing body of work (Table 3) looking at novel ways to transport cells at ambient/room temperature (RT) while attempting to maintain a high yield of cells at the point of delivery. With concerns growing regarding the clinical implications of cryopreservation during cell therapies, ambient shipping methods (paired with additional cell support modalities) may be a viable option. Ambient shipment completely negates the requirement of super-low temperatures (Figure 2); hence, it reduces logistical challenges, high costs, environmental impact, and the hazards associated with cryopreservation (Table 1). In the past, transporting cultures in medium-filled flasks at RT or ambient conditions has been employed as a transportation method.^106^^,^^123^^,^^128^ Nonetheless, this approach is restricted to short distances and durations, typically lasting up to 24 h, as numerous cell types fail to survive or thrive adequately during transit in cell culture flasks. This is primarily attributed to rapid oxygen and nutrient depletion, along with potential pH fluctuations.^106^^,^^129^

**Table 3:** Published articles regarding cell shipping at ambient temperatures

Study design	Study highlights	Effect on proliferation during ambient storage/shipment	Viability % (main endpoint)	References
Hypothermic preservation of adipose-derived MSCs (cultured in either fetal bovine serum [FBS] or human platelet lysate [HPL]-supplemented mediums) using BeadReady (Atelerix Ltd) encapsulation, while also comparing for **30 min, 5 days, or 12 days**^GEL^	MSC-FBS cell recovery showed a gradual drop throughout the time points (30 min: 71% ± 5%; day 5: 56% ± 5%; day 12: 44% ± 2%), while MSC-HPL appeared to show a more stable encapsulation profile with minimal cell loss within the first 5 days (30 min: 77% ± 5%; day 5: 77% ± 6%; day 12: 50% ± 5%) encapsulated MSC-FBS displayed a slight reduction in viability from 96% ± 0.3% (before encapsulation) to 76% ± 8% on day 12. Encapsulated MSC-HPL maintained high viability on day 12, ∼90%+. Post-encapsulation, recovered MSCs showed hematopoietic support capacity, robust differentiation potential, and expression of immunomodulatory molecules	not directly investigated during hypothermic storage; however, it was noted that HPL supplementation, which by itself improves MSC proliferation *in vitro*, did not negatively impact cell behavior during this encapsulation study	MSC-FBS: 76% ± 8% MSC-HPL: ∼90% +	Branco et al.^114^
Utilizing BeadReady (Atelerix Ltd) encapsulation to transport human limbus-derived stromal/MSCs at room temperature (RT) for **3–5 days** (in extreme Indian conditions)^GEL^	over a 3- to 5-day transit, the shipment container average temperature (RT) was 18.6°C ± 1.8°C, while the outside average ambient temperature was 31.4°C ± 1.2°C. Encapsulated cells subject to 3 days of RT or 4°C exhibited 82.45% ± 0.87% and 65.19% ± 1.19% viability, respectively. After 5-day transit, encapsulated cells exhibited 76.96% ± 1.98% (RT) and 64.45% ± 0.81% (4°C) viability. Non-encapsulated cells showed less than 5% recovery after 3–5 days (at both RT and 4°C)	not investigated	RT: 76.96% ± 2% 4°C: 64.5% ± 0.8%	Damala et al.^115^
Transporting human umbilical vein endothelial cells (HUVECs) without or with encapsulation (alginate ± carboxymethyl chitosan) at ambient temperature for up to **7 days**^GEL^	HUVEC-laden microcapsules were produced by an electrostatic spraying method. Alginate 1.5% [w/v] + 0.5% [w/v] carboxymethyl chitosan encapsulation increased the viability of HUVECS at 1, 3, 5, and 7 days when compared to non-encapsulated HUVECS. Similar viability results were found at both 4°C and 25°C. Encapsulated HUVEC survival increased as the carboxymethyl chitosan concentration increased from 0.1% to 0.5%	not investigated	4°C: 81.1% 25°C: 84.9%	Zhang et al.^116^
Using a novel medium (CellTravel) to transport or store MSCs at ambient temperature for **12–48 h**	CellTravel is an injectable solution made of phosphate saline buffer supplemented with amino acids and human serum albumin. ADSCs and UCMSCs exhibited high viability after storage at RT for 48 h, approximately 90% and 85%, respectively. Storage at 2°C–8°C significantly increased the viability of both cell lines after 48 h.	a significant increase in population doubling of ADSCs and umbilical-cord-derived MSCs was observed after 48 h at room temperature	RT: ∼85%–94% 2°C–8°C: ∼95% +	Kiet et al.^117^
Using a novel medium (CellShip) to transport cells at ambient temperature for at least **3 days**	after 72 h of CellShip ambient transport, HEK293, HepG2, CHO, K562, and Jurkat cells exhibited 88.2%, 96.2%, 97.4%, 97.8%, and 86.7% viability, respectively. Jurkat, HEK293, and HepG2 cell lines showed significantly higher metabolic activity when compared to cryopreserved controls	alamarBlue reduction (%) indicated increased proliferation of Jurkat, HEK, and HepG2 cells, following 72 h transport/storage in CellShip at ambient temperature compared with cryopreserved cells	86.7%–97.8%	Buick et al.^118^
Whole FBS as a cell protection reagent in the shipment of different cell lines (C3H10, 143B, and LX-2) at low or room temperature, for **3–10 days**	the optimal conditions for maintaining viability involved using a cell transport reagent with at least 50% serum, which successfully preserved the viability of C3H10, 143B, and LX-2 cells at temperatures ranging from 2°C to 16°C. Under these conditions, the cells remained viable for different durations: C3H10 cells for 3 days, 143B cells for 7 days, and LX-2 cells for up to 10 days; while retaining their primary characteristics after transportation	proliferation investigated after shipment but not during	80% +	Liu et al.^119^
1% alginate hydrogel beads used to transport cancer cells at ambient temperature between **5 days and 4 weeks**^GEL^	A549 lung cancer cells were shipped at room temperature for a duration of up to 5 days with no humidity or CO_2_ support. Viability on arrival was 60%–74.9%, and cells were effectively recovered and expanded in 2D culture after this. Note, shipment was carried out in warm climate (higher ambient temperature: 30°C–35°C).	culture of A549, HepG2, and U2OS cell lines with 3D alginate beads promoted proliferation after 8 days of ambient conditions	60%–74.9%	Alallam et al.^120^
Transporting embryonic stem cells in 100% FBS for **3–5 days** at ambient temperature	mouse ESCs demonstrated high viability and cell proliferation and a negligible effect on pluripotency-associated genes, after 3- to 5-day incubation at ambient temperature (4°C, 20°C, and 30°C), compared to dry ice	a cumulative increase in mESC cell number was seen after 5 days (cells almost doubled in number between days 4 and 5).	% not specified	Ye et al.^121^
Utilizing a “transporter” solution of 2% low-melting temperature agarose to transport cells at ambient temperature for **7 days**^GEL^	HeLa, NIH-3T3, RPE, and USOS cell lines exhibited high viability at 4°C, 20°C, and 37°C after 7 days in transporter (viability was considerably higher than dry ice methods). Effective growth was shown in HeLa and USOS cell lines 4 days after being kept in transporter for 3 days at ambient temperature	not thoroughly explored, but indicates that cell lines do not proliferate during transit	% not specified	Wheatley and Wheatley^101^
Effect of different temperatures (∼22°C or ∼7°C) and holding media on blastocyst development after **overnight** shipping	ViGRO embryo holding medium: *in vitro* maturation, cleavage, and blastocyst formation were significantly higher in 22°C room temperature (39%, 90%, and 41%, respectively) than 7°C (23%, 60%, and 17%, respectively). Similar observations were noted with SYNGRO holding medium; 7°C was detrimental to immature equine cumulus-oocyte complexes and their *in vitro* development	not investigated.	not assessed	Diaw et al.^122^
Mock shipment of mammalian cells at ambient temperature for **up to 7 days** using HemSol gel^GEL^	CHO, HEK293, and CACO-2 cells exhibited 92.2%, 81.2%, and 92.2% viability, respectively, after 7 days (HemSol gel, ambient temperature). After a 24-h recovery period at 37°C, primary cells (HUVEC, hepatocytes, MSCs, and B cells) exhibited 96.7%+ viability after 2–4 days of mock shipment (HemSol gel, ambient temperature)	not investigated	81.2%–96.7%	Stefansson et al.^123^
Three different anticoagulants (K3EDTA, sodium heparin, and citrate-based CPDA) were used to transport red blood cells (RBC) at 4°C and ambient temperature for **3 days**	RBC count and hemoglobin concentration did not change significantly within 72 h of simulated transportation at either temperature. Whereas RBC volume percentage, mean cell volume, and mean corpuscular hemoglobin concentration were only stable at 4°C. Most of the parameters except for ion (Na+, K+, Ca^2+^) handling and, potentially, reticulocytes count, tended to favor transportation at 4°C	not investigated	not assessed.	Makhro et al.^124^
Assessing different shipment temperatures and preservation media on mononuclear cell viability **over 2 to 7 days**	use of AQIX RS-I room temperature transport medium reduced the viability of PBMC and Jurkat cells during 2- to 7-day storage in the medium	not investigated	Jurkat cells—high viability: 90%+ PBMCs—high viability: ∼70%	Kofanova et al.^125^
Determining the effect of ambient temperature on populations of PBMC, CD8, and CD4 lymphocytes during blood shipment for **up to 24 h**	post-thaw, viability of PBMCs, CD8 lymphocytes, and CD4 lymphocytes stored at 15°C or 30°C for 2–12 h were not statistically different from PBMCs stored at RT (22°C) for 24 h	not investigated	∼60%–80%	Olson et al.^126^
An agarose-medium-gel-based method for cell transport for **3–5 days** at ambient temperature^GEL^	agarose concentration higher than 1.2% may reduce Madin-Darby canine kidney (MDCK) cell viability during ambient transportation	after plating equilibration (24 h at 37°C, 5% CO_2_), MDCK cells coated with 1% agarose slowly proliferated in the first 3 days (quadrupled in number) and plateaued between days 3 and 5 when exposed to ambient temperatures	% not specified	Yang et al.^127^

Study highlights and the effects on proliferation and viability are summarized. Not an exhaustive list (^GEL^: indicates use of gel structural support).

Temperature fluctuations are a major limiting factor of ambient cell transport (Figure 2). However, evidence exists to suggest cells remain viable and functional at ambient temperatures. An investigation by Hunt et al. found that after paused culture, recombinant Chinese hamster ovary cells (CHO-Clone 161) exhibited high cell viability and exponential growth in a temperature range of 6°C–24°C for up to 3 weeks (suspended in culture medium in microcentrifuge tubes, absent of CO_2_). At 6°C, CHO-Clone 161 viability was 60% on day 6 of storage. At a lower temperature of 4°C and an extended period of 9 days, CHO-DG44 and human embryonic kidney (HEK293 EBNA) cells resumed exponential growth when incubated at 37°C. This study showed that with limited additional cellular support (only suspended in medium), cells still exhibited robust recovery and viability at lower temperatures.^130^ Wang et al. explored cell shipment and varying ambient temperatures on cells suspended in culture medium: 293T, Saos2, K562, and HeLa cells subject to 16°C–22°C for 4 days exhibited high viability, approximately 80%. Interestingly, at 5°C HeLa cells exhibited approximately 50% viability on day 4. Primary mouse embryonic fibroblasts and mouse embryonic stem cells (mESCs) exhibited approximately 70% and 40% viability, respectively, after 4 days of 16°C–22°C incubation. Melanoma cells retained the same cell density or higher after 4 days of culture at 5°C–22°C. Shipment of cells from Manchester (UK) to Wuhan (China) at ambient temperature (approximately 36-h transit time) yielded high viability of melanoma (∼65%), 293T (∼80%), Saos2 (∼85%), and mESCs (∼60%). Colony formation ability was also observed. Conversely, cells shipped with ice packs yielded significantly lower viability and colony-forming ability.^128^ Viability of human-adipose-derived stem cells (hASCs) significantly varied when preserved in alginate under hypothermic temperatures (4°C–23°C).^99^ Encapsulated hASCs stored at 15°C for 72 h yielded the highest recovery rate of 86% ± 6%, significantly higher than control samples. Below or above 15°C, viability slightly decreased, but a significant decrease was observed at 23°C, with a recovery rate of 29% ± 29%. Consistent viability above 70% was achieved at temperatures between 13°C and 19°C, meeting FDA viability standards for clinical translation.^99^ Table 3 summarizes published papers investigating cell transport at ambient temperature. Briefly, it is important to note that when cells are being transported at ambient temperature, they have the potential to expand. In most cases, lower culture temperatures (e.g., ambient temperature exposure/cold stress) usually suppresses proliferative capacity of a wide variety of cell lines.^131^^,^^132^ However, some studies (see Table 3) highlight significant increases in cell number during ambient storage/shipment. Future studies would need to consider proliferation during ambient transport, as it could dictate and/or affect (1) cell loading density, (2) nutrient consumption, (3) oxygen consumption, (4) 3D structural support considerations, and (4) cell over-crowding, subsequently potentially affecting cell morphology, function, and viability.

## Considerations for effective ambient cell transport

Ultimately, if cells can be transported at ambient temperatures, it will reduce the number of manipulations at the point of delivery (Figure 2). For example, in a clinical setting, CPAs will be absent and hence cells do not have to be washed or subcultured, whereas in a research setting it means cells can be used almost immediately in experiments. Ekpo et al. recently highlighted options to avoid DMSO in biotherapeutics, ultimately avoiding impaired cellular function and DMSO toxicity in patients.^133^ It is clear that the scientific community is urging for new options to avoid CPAs and cryopreservation in some cell-based therapies. We strongly encourage the exploration of novel ambient cell transport systems. However, to transport cells at ambient temperature, additional cell support measures need to be considered, primarily in the form of oxygen, nutritional, and structural support (Figure 3).

### Oxygen support

In recent years, focus has been shifted to incorporate an oxygen supply to cells during cell transport and cell therapy processes.^134^^,^^135^^,^^136^ Inadequate oxygen supply (hypoxia) can impede cellular function (e.g., reduced metabolism, promote senescence, and changes in cell functionality), which could potentially affect experimental and clinical procedures. If prolonged cell transport at ambient temperatures is to succeed, oxygen supply must be considered—to date, and to the best of our knowledge, we cannot find published work that has incorporated an oxygen supply to help elongate the shelf life of live cell transport. However, we have unearthed several studies that have devised “oxygen-releasing” constructs for cell maintenance, with a large amount of work being carried out on pancreatic islet cells (refer to Table 4). These examples may be considered to help incorporate oxygen supply during live (ambient) cell shipment (Figure 3). Some of these technologies (Table 4) include (1) “oxygen-generating” materials (usually peroxides that can generate oxygen *in situ* by reaction with water), (2) “oxygen-transporting” materials (materials like perfluorocarbons that can solubilize and transport high quantities of oxygen), and (3) external oxygen delivery (devices that can infuse exogenous oxygen). These technologies have been reviewed by Goswami et al. with a focus on work being carried out on pancreatic islet cell transplantation.^134^ Therefore, these technologies could potentially be adapted for ambient cell transportation. Moreover, as these technologies were initially designed to increase cell survival in tissue engineering applications, they would have already considered some of the potential limitations of the materials used, including sterility, degradation rates, or regulatory concerns. A relevant example of this is the use of perfluorocarbons as “oxygen-transporting” materials, which were previously used as artificial blood substitutes in FDA-approved products (Fluosol-DA, OptisonTM, etc.^145^) and hence have already circumvented some of the potential translational limitations that oxygen supplying systems for ambient cell transportation could encounter. Table 4 highlights some oxygen support considerations for live cell shipment.

**Table 4:** Oxygen support considerations for live cell shipment—potential ways to generate an oxygen supply to live cells during ambient cell transport (not an exhaustive list)

Cell support system	Summary and outcomes	References
A novel inverse breathing encapsulation device (iBED)—CO_2_ released from respiring islets and cells in the host tissue is recycled into O_2_ by lithium peroxide (Li_2_O_2_)	>CO_2_-responsive O_2_ release was observed, which improved cell survival in hypoxic conditions >iBEDv3 design enabled 3-month diabetes correction in STZ-induced diabetic C57BL6/J mice *via* subcutaneous implantation (normoglycemia was achieved in 8 of 10 iBEDv3-treated mice for 92 days) >iBEDv3S was able to notably improve islet cell survival and function in a xenogeneic subcutaneous transplantation in large animals (minipigs). Compared to control devices, insulin expression was 9.6-fold higher, and DAPI content was 3.4-fold higher in islets from the iBEDv3S devices, indicating improved function and survival/viability	an inverse-breathing encapsulation system for cell delivery^137^
A novel hyaluronic acid/perfluorocarbon biomaterial (Oxygel) for prolonged oxygen release to INS-1E- and PSC-derived β-cells (modeling and experimental validation)	>Oxygel exhibited shear thinning and self-healing properties >Oxygel can carry high O_2_ payloads and slowly release them for 90 h, exhibiting 14.5 times smaller O_2_ diffusivity than PBS >modeling predicted the oxygen durability within Oxygel upon cell encapsulation and was experimentally validated *in vitro*	a predictive oxygen durability model to analyze oxygen consumption of insulin producing cells encapsulated within a highly oxygenated hydrogel^135^
Utilizing a hydrolytically reactive oxygen-generating material in the form of polydimethylsiloxane (PDMS) encapsulated solid calcium peroxide (OxySite) for the treatment of type 1 diabetes	>compared to control devices, after 72 h, a 1.5-fold increase in (MIN6) beta cell metabolic activity (MTT) and a 1.7-fold increase in the insulin stimulation index were observed for OxySite loaded constructs > OxySite also boosted pancreatic rat islet metabolic activity (MTT, 1.83-fold higher) and function (insulin content, 1.97-fold higher) within microencapsulation device under hypoxic conditions (48 h) > OxySite enhanced the efficacy of beta cell macroencapsulation devices in an immunocompetent diabetic mouse model (100% of recipients achieved euglycemia and 50% achieved normoglycemia) > OxySite macroencapsulation constructs supports human islet survival at elevated loading densities – increased metabolic activity and glucose stimulation index was observed	oxygen-generating biomaterial improves the function and efficacy of beta cells within a macroencapsulation device^138^
Improving stem cell survival (and tissue regeneration in ischemic hindlimbs) via oxygen-releasing microspheres	>microspheres are capable of releasing oxygen in response to environmental oxygen level (a potentially safer approach to oxygen-releasing biomaterials) >released oxygen significantly enhanced MSC survival without inducing ROS production (over 28 days) under hypoxic condition > co-delivery of microspheres and MSCs to the mouse ischemic limbs ameliorated MSC survival, proliferation (increased Ki67^+^ cells) and paracrine effects (increased bFGF, PDGF-BB and HGF) under ischemic conditions (after 28 days). >significantly accelerated angiogenesis, blood flow restoration, and skeletal muscle regeneration (increased muscle fiber diameter) without provoking tissue inflammation was observed after 28 days post-delivery of oxygen-release microspheres and MSCs.	oxygen-releasing microspheres capable of releasing oxygen in response to environmental oxygen level to improve stem cell survival and tissue regeneration in ischemic hindlimbs^139^
A biomimetic scaffold featuring internal continuous air channels which facilitates rapid O_2_ transport through the whole system to cells, named SONIC (Speedy Oxygenation Network for Islet Constructs)	>SONIC indicated a 10,000-fold higher O_2_ diffusivity than hydrogels >SONIC facilitates rapid O_2_ transport through the whole system to INS-1 cells several millimeters away from the device-host boundary (incubated in a hypoxic incubator with 5% CO_2_ and 5% O_2_, for 48 h) >*in vitro* results were consistent with computational modeling. > rat islets (500 IEQ per transplant) were incorporated in the SONIC devices (or controls) and transplanted into the intraperitoneal cavity of diabetic C57BL/6 mice -the SONIC device enabled 6-month diabetes correction in mice >after 6 months, viable islet cells were retrieved from mice	a bioinspired scaffold for rapid oxygenation of cell encapsulation systems^140^
Utilizing a hydrolytically activated oxygen-generating biomaterial in the form of polydimethylsiloxane (PDMS)-encapsulated solid calcium peroxide (PDMS-CaO_2_) to support survival of β cell line and pancreatic rat islets	>during the first 2 weeks, PDMS-CaO_2_ disks generated oxygen (0.12–0.16 mM) to a degree that results in the shift from hypoxia to that close to optimal cell culture conditions >PDMS-CaO_2_ disks mitigated hypoxia-induced MIN6 (24 h) and pancreatic islet cell (48 h) death as shown by increased MTT as well as reduced LDH release and caspase activity > PDMS-CaO_2_-disk-induced enhanced and sustained MIN6 cell survival over 3 weeks was observed under low oxygen conditions >significant increase in viability of MIN6 cells within 3D agarose constructs containing PDMS-CaO_2_ disk (after 3 days)	preventing hypoxia-induced cell death in beta cells and islets via hydrolytically activated, oxygen-generating biomaterials^141^
An *in situ* electrochemical oxygen generator that utilizes electrolysis to generate oxygen from water to help nourish encapsulated cells	>growth and viability of βTC3 cells improved with oxygen generation >the highest cell viability was observed in devices cultured in stirred media and O_2_ generation (over a 3-day period, inclusive of alginate)	*in situ* electrochemical oxygen generation with an immunoisolation device^142^
photosynthetic oxygen supply from unicellular alga *Chlorella* to co-encapsulated islets in alginate and perifused with oxygen-free medium	>no insulin response to glucose was obtained upon inactivation of photosynthesis by darkness >islets co-encapsulated with alga cells and placed in an anoxic environment subject to illumination showed a statistically significant increase in insulin secretion in response to secretagogues (compared to encapsulated islets alone)	photosynthetic oxygen generator for bioartificial pancreas^143^
Islet cells immobilized in an alginate slab were mounted on a photosynthetic slab whereby oxygen is produced photosynthetically by microorganisms (*Synechococcus lividus*)	>cells and photosynthetic component are separated by a gas-permeable silicone rubber-Teflon membrane (immune protection) >upon illumination, photosynthetically produced oxygen diffused via the silicone Teflon membrane into the islet compartment - oxygen production was stable for 1 month. >implantation of device into STZ-induced diabetic rats saw blood glucose concentrations rapidly dropped to normoglycemic levels. Following device explantation (7 days later), blood glucose concentrations returned to the diabetic range	oxygen supply by photosynthesis to an implantable islet cell device^144^

### Nutritional support

Cells are metabolically active; hence, they require nutrients and oxygen to survive. Similarly to cells being cultured in a laboratory setting, cells being transported at ambient temperature will require nutritional support (Figure 3), usually within a basal medium.^16^ Fetal bovine serum (FBS) and other serum-based nutritional solutions are commonly considered for live cell transport as they are rich in nutritional provision—key components of commercial FBS include serum-associated proteins, hormones, growth factors, cytokines, fatty acids, lipids, carbohydrates, nonprotein/nitrogen, vitamins, minerals, and inorganic compounds. FBS constitutes over 100 nutritional compounds that can be utilized by cells.^146^ More recently, research has been carried out to reduce serum dependency in cell culture.^147^ Studies have shown that optimized nutritional support can bolster cell development and survival. For example, Lyra-Leite et al. showed through rigorous optimization how nutritional support can be reduced (to just 39 components) to effectively support human iPSCs.^148^ Studies like this highlight that oversaturation of nutrients is not always required for effective growth and how optimized nutrient formulations can effectively support cell survival. Work like this can be translated to ambient cell shipping methods to develop cell-specific nutritional support systems. However, in a clinical/cell therapy setting, a very important consideration is to ensure the nutrient source is xeno-free, so the recipient is not at risk of contaminants, diseases, or an immune response. Furthermore, utilization of xeno-free products in cell therapeutics has been shown to improve immunomodulatory properties,^149^ provide a consistent reproducible environment (improved biological consistency) and reduce the risk of viruses and prions.^150^

### Structural support

Biological products are prone to various stresses during shipment, most notably shock movements and vibrations (caused by turbulence and handling). Hence, structural support for biological materials/cells must be considered (Figure 3). Studies have monitored vibrations during land and air (Europe to USA flight) delivery transport.^151^^,^^152^ Shock pulses of 100 ms durations with peak accelerations of 20 or 50 ms^−2^ were reported during land transport and shock pulses lasting 20 ms, with a maximum peak acceleration of 850 ms^−2^ during air transport. Data from these studies were used as a guide to assess the effect of vibrations on human MSCs. Although no striking changes in MSC viability were observed following vibration treatment, vibration at 25 Hz (peak acceleration 140 ms^−2^ and peak displacement 5.7 mm) for 24 h increased CD29 and CD44 expression.^153^ In another study, a vibration frequency of 100 Hz significantly increased neurite density and 40 Hz increased cell proliferation and neurite length of SH-SY5Y cells.^154^ Here are some indicators that the effects of transport could affect cell function and hence should be considered when designing live cell transport systems.

Hydrogels, 3D structures composed of crosslinked hydrophilic polymers (thus retaining water), offer high potential in providing support and protection to cells during transport. A major benefit of hydrogels is they exhibit permeability to oxygen and nutrients, as well as small molecules, e.g., cytokines.^106^^,^^155^ Furthermore, hydrogels are unique in the fact that they can be engineered to have controlled porosity, mechanical stability, topology, topography, viscoelasticity, and immunoregulatory properties.^156^ Due to their viscoelastic properties, hydrogels can also dampen the effects of (micro)vibrations.^157^^,^^158^^,^^159^ These characteristics fit perfectly with the idea of combining oxygen- and/or nutrient-providing systems with hydrogel encapsulated cells for ambient transport. Ultimately, choosing an effective structure to encapsulate cells can support cell morphology and function while also offering a protective and supportive environment (Figure 3). Subsequently, this design can be modified, so the environment can be appropriately enriched with oxygen and/or nutrients. Commonly used hydrogels include agarose, gelatin, alginate, HA, collagen, polyacrylamide, and polyethylene glycol.^106^^,^^160^ Due to the vast array and configurations of hydrogels, the next section will explore this topic in more detail, highlighting the potential of hydrogels to drive live ambient cell shipment.

Although it is difficult to accurately estimate the cost behind scaled-up ambient cell transportation, we anticipate that ambient transportation systems would be more cost-effective than cryo-transportation. To illustrate this, a rough estimation of the cost (USD) of providing nutrient, cell, and oxygen support to a hydrogel system can be explored—Oxygel is as an example in which both oxygen and structural support are provided by the gel formulation.^135^ Therefore, cost would depend on (1) the material cost of Oxygel formulation: 1% w/v HA is approximately 1$–60$/g (depending on grade)^161^ and 28% w/v perfluorodecalin is approximately 2 $/g,^162^ and (2) the cost of oxygenation and the oxygen needed: 30 L of O_2_ per mL of Oxygel, with an estimated O_2_ price of 0.005$–0.08$/L. Considering this, it could be estimated that the added price of using Oxygel for ambient transportation would be around $2 to transport 1 × 10^6^ cells/mL (0.8$/mL for material formulation and 1.2$/mL for material oxygenation). This represents a relatively small expense compared to the high prices behind cold chain transportation, with some companies like ARK Cryo offering cryo-shipments of stem cells ranging from $1,850 to $5,220 per shipment, depending on the route.^163^

## Hydrogels for cell encapsulation—a pivotal player in ambient cell shipment development

**Figure 4:**
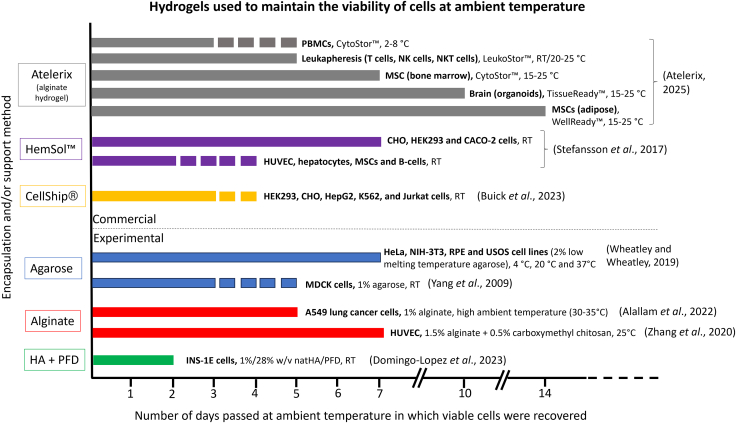
Examples of commercial and experimental ways to utilize hydrogels for ambient cell shipment or ambient cell encapsulation For each example, the number of days passed in which a high proportion of viable cells were recovered is shown. Dotted lines indicate periods where there is a drop in viability (or insufficient data). Cell lines are indicated in bold, followed by hydrogel formulation and transport temperatures tested. Some commercial products do not disclose their hydrogel formulations or viability data. RT, room temperature; natHA, natural hyaluronic acid; PFD, perfluorodecalin. References included:^101^^,^^116^^,^^118^^,^^120^^,^^123^^,^^127^^,^^135^^,^^175^.

A major avenue of research to help support ambient cell shipment development is hydrogel incorporation. Hydrogels are 3D structures formed by the physical or chemical crosslinking of polymers, which can retain large amount of water (typically >90%) within their matrix.^164^ The excellent biocompatibility and highly tunability of hydrogels allows them to be designed to mimic the mechanical properties of a target tissue,^165^ by mimicking their extracellular matrix (ECM). Several hydrogels or scaffolds based on naturally occurring (collagen, HA, etc.) and/or structurally similar (alginate, agarose, etc.) ECM components have shown improved survival and function of cells on their own^166^^,^^167^^,^^168^^,^^169^ or when incorporated within encapsulation devices.^170^^,^^171^^,^^172^^,^^173^ Due to their ability to provide structural support and offer a porous and diffusible environment and their versatility (natural vs. synthetic, stiffness, matrix-interactions, etc.),^174^ hydrogel-based systems have been used to encapsulate and transport various cell types, serving as an alternative to cryopreservation (refer to Figure 4). Some of these systems include agarose, gelatin, collagen, alginate, HA, etc., which have been used in a range of temperatures from hypothermia (4°C) to RT (Table 3). These systems are especially relevant for the transportation of stem cells, especially considering that stem cells in suspension culture do not usually survive long periods of transportation at RT,^176^ and exhibit some negative effects after cryopreservation mainly due to DMSO toxicity.^177^

### Agarose

Agarose is a polysaccharide derived from seaweed that, in the presence of water, can create gel matrices/beads. These scaffolds can be used to encapsulate cells due to their porous structure that allows for nutrient and gas exchange, while providing support for adherent cells, promoting cell viability and growth.^178^ Rigidity plays a vital role in supporting adherent cells and preventing cell death; thus, the agarose-medium gel must possess appropriate stiffness. Concentrations below 0.8% lack sufficient hardness, while concentrations above 1.2% result in dry and fragile gels, potentially compromising cell viability. Therefore, it was found that a concentration of 1% agarose achieved the best viability for the encapsulation and preservation of various cells of different nature, including MDCK, HEK-293, and human lung adenocarcinoma epithelial (A549) cells.^127^ Agarose-medium gels were also able to keep the morphology and function of these cell types intact under “simulated-transport” conditions (placed at RT for 3–5 days). In a different study, an agarose-gel-based method, based on a supplemented 2% solution of low-melting temperature agarose, demonstrated the ability to transport cultured cells without dry ice for up to 2 weeks.^101^ A range of cryo-sensitive cells were evaluated, including untransformed NIH 3T3 mouse fibroblast RPE cells (human retinal epithelial) or transformed cell lines (HeLa, A549, etc.). The authors evaluated different shipping temperatures, ranging from hypothermia (0°C–4°C), controlled RT (20°C–22°C), and warm ambient temperatures (27°C, 32°C, and 37°C), as well as cell densities. Despite some differences found in the viability under different conditions, it was found that concentrations of 5 × 10^6^ cells/mL transported at temperatures between 4°C and 27°C ensures optimal recovery upon arrival at the destination (Figure 4).

### Collagen and gelatin

Collagen and gelatin, derived from animal tissues, are protein-based materials renowned for their biocompatibility and ability to form scaffolds that mimic ECM. These scaffolds provide an ideal microenvironment for cell attachment and growth, while also controlling cell communications and signaling *via* interactions with integrins and maintaining cellular homeostasis,^179^ and hence can be used to improve cell survival during transportation. For instance, the biocompatible and porous Optimaix-3D scaffold, manufactured from collagen, demonstrated its ability to transport primary human hepatocytes (for 24 h at 37°C) and maintain their viability, essential hepatic functions, and metabolism for over 10 days post-transport,^180^^,^^181^ outperforming cells sent in suspension cultures (2D) following the same shipping.^181^ Additionally, scaffold cultivation in Optimaix-3D cultures can significantly impact the metabolic functions of cells, such as urea production and albumin synthesis, providing more stable and enhanced cellular functions over time.^181^ Alternatively, gelatin, derived from the partial hydrolysis of collagen, serves as a versatile biomaterial in fabricating cellular scaffolds with improved processability, tunable properties, and lower risk of immunogenicity. HemSol, a combination of gelatin (10%) with a mix of osmolyte sugars (trehalose, mannitol, glucose, and dextran), was shown to be a viable alternative to dry ice for cell shipping, suitable for both surface-adherent and free-floating cell cultures at ambient temperatures.^123^ HemSol gel resulted in high cell viability after a mock transportation for up to 7 days at ambient conditions (Figure 4). Compared to dry ice shipping, cells shipped in HemSol gel exhibited over 95% viability and could recover biological activity within 2 h post-transit, eliminating the need for washing steps to remove DMSO.^123^

### Alginate

Alginate hydrogels, made of polysaccharides derived from brown seaweed, offer several advantages for ambient transportation, including biocompatibility and tunable mechanical properties, as well as ease of gelation into different conformations, such as gels, films, beads, etc. The mechanical properties of alginate hydrogels, influenced by polysaccharide and cationic cross-linker ratios, can be optimized using calcium, strontium, and barium as cross-linking ions for enhanced stability.^182^ Studies involving human (h)MSCs and mESCs encapsulated and stored in alginate hydrogels (4.8% w/v concentration) demonstrate high cell-survival rates and comparable proliferation and protein levels compared to cryopreservation methods. Human mesenchymal stem cells (hMSCs) and mESCs showed a viability of 80% and 74%, respectively, post-extraction from alginate hydrogels stored for 5 days under ambient conditions in an air-tight environment. Encapsulation of these cells in alginate showed comparable survival and proliferation rates and protein expression levels (OCT3/4, SOX-2, SSEA, etc.) that were comparable to cryopreserved cells with 10% DMSO.^100^

Alginate microencapsulation has emerged as a sophisticated methodology to enhance the survival of cryopreserved therapeutic cells by reducing post-thaw apoptosis. As a result of this, significant enhancement in post-thaw cell viability has been achieved using alginate microcapsules, attributed to the preservation of crucial cell-cell and cell-matrix interactions.^183^ This was demonstrated for the cryopreservation of hESCs, showing high proliferation rates and cell recovery yields (<70%) when cryopreserved in DMSO-free alginate microencapsulates (1.1% concentration), which was three times superior compared to non-encapsulated cryopreserved hESCs. Similarly, human adipose-derived stem cells (hASCs) were encapsulated in alginate (1.2% w/v concentration) and preserved for 3 days at a range of temperatures, ranging from hypothermia to RT (4°C–23°C).^99^ Alginate encapsulation demonstrated temperature dependency in cell viability, with an optimal temperature of 15°C (86% ± 6%) at all other temperatures except 4°C and 23°C, achieving a viability higher than 70% and superior to non-encapsulated cells. Due to the promising benefits of alginate systems in preserving and transporting cells at hypothermic or ambient temperatures, companies like Atelerix have emerged, offering alginate-based kits for the storage and transportation of cells in suspension (BeadReady), plated (WellReady), and even living tissue samples (TissueReady). Atelerix provides a guide of recommended storage temperatures and maximum testing time of encapsulations for different cells lines, e.g., HEK293 cells can be stored for up to 10 days at 15°C–25°C (RT) using Atelerix CytoStor (additional examples can be found in Figure 4).^175^

### Hyaluronic acid

Although alginate, collagen, and gelatin systems hold great potential, some incorporate components not naturally found in humans or involve complex and tedious cell isolation procedures (e.g., in alginate hydrogels/beads, electrostatic crosslinking needs to be broken by chelating agent for cell extraction). Therefore, an interesting alternative for cellular therapies would be the use of naturally occurring hydrogels, such as HA, which can be injected and/or implanted after ambient shipment without the need of cell isolation or rescue procedures. HA is a polysaccharide of the family of the glycosaminoglycans (GAGs), which is naturally synthesized by transmembrane hyaluronan synthases and extruded into the extracellular space, producing polymer chains of different lengths, ranging from 1 × 10^5^ to more than 2 × 10^6^ Da.^184^^,^^185^ To maintain their homeostasis, HA is naturally degraded by hyaluronidases and/or by the effect of ROS.^185^ GAGs (including chondroitin sulfate, heparan sulfate, and HA, which is the most abundant) are key components of the ECM, as they are responsible for most of their physical functions such as providing mechanical support for cells, maintaining hydration of tissues, and modulating diffusion and exchange of biomolecules and/or ions. Moreover, HA has been found to be key for modulating cellular behavior.^186^ Currently, there exists a variety of HA hydrogel products in the clinics for a wide range of applications ranging from wound healing, cosmetic, ophthalmologic and gynecologic, and more in the pipeline.^187^ HA hydrogels, such as HyStem, have been extensively used in 3D cell culture, especially with stem cells as they can replicate more accurately the *in vivo* environment, assisting their survival, differentiation, and functionality.^188^^,^^189^ In the context of cell preservation and shipment, HA hydrogels have shown to exhibit a cytoprotective effect during cryopreservation, making it possible to reduce DMSO concentration from 10% to 3% by including 0.1%–0.2% HA in hMSC storage protocols.^190^ Particularly, it was found that using a combination of 3% DMSO and 0.1% high-molecular-weight HA (>1 MDa) resulted in higher post-thaw recovery, increased proliferation (corelating with an increase in the expression of stemness marker CD49f), maintained viability (>75%), and unchanged differentiation capacity compared to controls. Despite these promising results, to date, there are no readily available systems designed for the ambient transportation of cells using HA hydrogels.

Something to consider when designing a system to transport therapeutic cells in ambient conditions would be the nutritional and oxygen requirements of the cells during transport (as discussed in previous sections; Figure 3). Regardless of *in vivo* or *in vitro* conditions, cells require nutrients and oxygen to survive and function effectively. Hydrogel platforms offer the opportunity to address these needs. First, due to the high water content of hydrogels, hydrogel scaffolds can be formulated with cell-specific supplements in order to improve the cellular performance of the encapsulated cells.^135^^,^^160^^,^^191^ This could expand the opportunities of cell transport to different cell lines, as the hydrogel formulation could incorporate cell-specific supplements, taking into account the metabolic requirements of the cell transported and potentially increasing their survival during transit. With respect to oxygen, several strategies have been investigated to provide oxygen to cells *in vivo*, including external oxygen delivery, oxygen-generating materials, or oxygen-transporting materials (refer to Table 4).^134^ Although these technologies have been initially designed to overcome hypoxia *in vivo*, for instance in cell transplantation applications for bioartificial organs, they could also be applied to ambient cell shipment.^134^ In this context, an interesting material is Oxygel, which combines an ECM-mimicking HA hydrogel (formulated with cell-specific FBS-free cell culture media) with perfluorocarbon-lipid nanoemulsions that have the ability to transport and provide oxygen to encapsulated cells for prolonged periods of time (more than 90 h).^135^ In addition, oxygen consumption during transit should be considered and adjusted for each specific cell line—Domingo-Lopez et al. showed that an oxygen durability model could be used to predict oxygen release over time from oxygenated hydrogels, based on the cell-specific metabolic requirements (in this case, the oxygen consumption rate) and cell density.^135^ This model, which was proven with insulin-producing cells (INS-1E), could be tailored to different cell lines helping to define the time frames for successful ambient transportation. Technologies like this could potentially provide additional benefits to maintain cell survival during ambient transport, especially to clinically relevant hypoxia sensitive cells such as β-cells and neuronal cells.^192^^,^^193^

## Summary

It is evident that the current protocols utilizing cryopreservation to store and ship cells for FDA-approved cell therapies are effective in eliciting a clinical response and ameliorating the target disease state. However, there are some drawbacks associated with cryopreserved cell therapies, ranging from (1) financial/socioeconomic: cell therapies cost an astronomical amount to the average recipient, e.g., for some CAR T cell therapies, the average price range for treatment is approximately USD $373,000–711,884.^194^ Moreover logistical/supply chain costs associated with cryopreserved (CAR T) cell shipment makes up approximately 30% of the total cost.^195^^,^^196^ A study that explored vaccination shipping methods showed that switching from cold chain to ambient temperature transport for up to 4 days reduced the cost per unit by 50%.^197^ If this example was translated to cell therapy transportation, it could lead to the saving of tens of thousands of dollars; however, this is only a proxy measure, and other factors would have to be considered when dealing with modern cell therapy transport; moreover, this example provides an indicator of the potential financial savings of ambient transport in the medical world; (2) cell viability upon patient infusion: although FDA guidelines state cell viability must reach a specific threshold before infusion, there is evidence that this quota is sometimes missed (as low as 47.2%), an effect that is influenced by the cryopreservation process^53^; and (3) the yet-unknown *in vivo* effects of infused thawed cells during cell therapies: for example, it is still unknown whether infused cryopreserved cells may potentially induce negative effects on patients in the long-term.^198^

With increasing demand and growing interest from pharmaceutical companies, the manufacture of cell therapies (especially for cancer, i.e., CAR T therapy) has integrated automated and cryopreservation methods to accelerate and streamline the generation of cell-based therapies. Cryopreservation techniques have been incorporated into cell therapy manufacturing as many cell therapies are generated in specialized facilities away from a hospital setting and hence products need to be safely stored and shipped. On their own or in combination, automated and cryopreservation techniques may dampen the clinical potency of cell therapies by reducing the viability of infused cells. Moreover, increased cell numbers are required to account for cryopreservation-induced cell death.^56^ Most studies have reported an appropriate number (70–80%+) of viable cells being administered to patients. However, the exact number of viable cells at the point of infusion (post-thaw if cryopreserved) has not been well documented. Therefore, the exact *in vivo* short- and long-term effects of cell therapies exhibiting reduced viability are currently unknown. It is evident that for a large proportion of patients receiving cell therapies, effective treatment is provided regardless of viability 80% or slightly lower; moreover, it is not known how much more effective cell therapy could be if cryopreservation is avoided, and subsequently high viability is maintained (≥95%). With the exponential increase in cell therapies and their automation, more work is required to assess and address the effects of reduced viability following manufacture—exploring *in vivo* CIDOCD of CAR T and other prominent cell therapies is highly encouraged. In an attempt to avoid the negative effects of cryopreservation on some cell types and cell therapies, novel transport solutions should be explored, especially ambient-based systems.

### Ambient cell shipment—future considerations

Ambient shipment of cells could be an effective tool during cell therapy processes, as it would avoid exposure to CPAs and the cryopreservation process, minimize or potentially avoid cold chain logistics, offer immediate use (e.g., direct patient infusion), and potentially significantly reduce the financial burden associated with cold chain transport (Table 1). Fresh, non-cryopreserved cells could offer more efficacious treatment potential for existing and developing cell therapies (e.g., islet cell and hepatocyte transplantation); hence, ambient transport methods could be pivotal in actualizing this idea. Also, a more affordable and less hazardous (no liquid nitrogen or dry ice) cell shipment option will open new avenues of clinical and experimental investigations in more economically disadvantaged and rural nations or communities. With all these potential benefits, more research is warranted regarding ambient cell shipment.

We encourage the integration and optimization of three major elements—an oxygen reservoir, nutrient supply, and structural support (e.g., *via* hydrogel incorporation; refer to Figure 3). Different configurations of these three elements could theoretically support cellular viability and function at ambient temperatures, making it possible to adjust the ambient transportation systems to the metabolic and cell support demands of different cell lines, as demonstrated by Domingo-Lopez et al. using Oxygel (where the formulation was adjusted with the specific nutrients and oxygen supply required for the cell encapsulated).^135^ The beauty of having different adaptable components in an ambient transport device is that it can be modified and optimized for a wide range of cells and applications. For example, for oxygen-sensitive cells (e.g., pancreatic beta cells), a larger oxygen reservoir can be provided, whereas metabolically active cells (e.g., hepatocytes and myoblasts) could be provided with a richer nutritional source. Furthermore, if cells arrive at their intended destination without the requirement of manipulations, it could potentially offer the opportunity to directly infuse cells into a patient. This could be highly beneficial in a setting where encapsulated cells are required to be engrafted into a target site. As seen in Figure 4 and Table 3, ambient shipment methods (in conjunction with hydrogel support systems) have demonstrated successful cell shipment over varying time frames (up to 14 days). We recommend that ambient cell shipment methods should aim to maintain cell survival for approximately 5 days, subsequently allowing for national and potentially international transport, as well as mitigating some logistical hold-ups.

Cell therapies are evolving rapidly, and in less than a decade, the cell therapy market is expected to generate over USD $25 billion.^33^ We must evolve with them, and we must explore the potential of ambient cell shipment while ultimately ensuring patients receive potent, viable, and efficacious cell-based treatments. Fresh, non-cryopreserved cell products could give way to a broader range of cell therapies to treat more diseases, which without ambient transport would never be possible.

## Acknowledgments

This publication has emanated from research conducted with the financial support of Taighde Éireann – Research Ireland’s National Challenge Fund under grant number 22/NCF/DR/11293 and Taighde Éireann – Research Ireland under grant number 13/RC/2073_P2 at CÚRAM Research Ireland Center for Medical Devices. D.A.D.-L. was funded by the European Union’s Horizon Europe research and innovation program under the Marie Skłodowska-Curie grant agreement No 101153515.

## Author contributions

J.G. conceived and designed the review, conducted the literature review, and wrote the initial draft of the manuscript. D.A.D.-L. conducted the literature review, contributed to the writing of the initial draft, and participated in the revision of the manuscript. J.G., D.A.D.-L., R.E.L., R.T. and G.P.D. provided critical revisions and participated in the final editing of the manuscript. R.E.L. and R.T. acquired funding. All authors approved the final version of the manuscript.

## Declaration of interests

The authors declare no competing interests.
